# Supplementary material for: Glucokinase Variant Proteins Are Resistant to Fasting-Induced Uridine Diphosphate Glucose-Dependent Degradation in Maturity-Onset Diabetes of the Young Type 2 Patients
Source: Int J Mol Sci. 2023 Oct 31;24(21):15842. doi: 10.3390/ijms242115842 (PMC10649437; doi:10.3390/ijms242115842)
Supplement: Supplementary file 1 [file ijms-24-15842-s001.zip › ijms-2650363-supplementary.pdf]

Supplemental information for

Glucokinase variant proteins are resistant to uridine diphosphate glucose-dependent degradation in maturity-onset diabetes of the young type 2 patients.

This pdf file includes:

Abbreviations

Supplementary Figures S1 to S3

Clinical data

Abbreviations

BSA, bovine serum albumin

cIAP1, cellular inhibitor of apoptosis protein 1

CRBN, cereblon

CRL4, cullin ring ubiquitin ligase 4

CUL4, cullin 4

DDB1, damage-specific DNA binding protein 1

ELISA, enzyme-linked immunosorbent assay

ER, endoplasmic reticulum

FIRI, fasting immunoreactive insulin

FCS, fetal calf serum

FPG, fasting blood glucose

GCK, glucokinase

G1P, glucose-1-phosphate

G6P, glucose-6-phosphate

GLUT, glucose transporter

GSIS, glucose-induced insulin secretion

HOMAβ, homeostatic model assessment of β-cells

HOMA-IR, homeostatic model assessment for insulin resistance

HEK293T, Human embryo kidney 293T

IC_{50}, 50% inhibitory concentration

IMiDs, Immunomodulatory drugs

IP, immunoprecipitation

KD, knockdown

MDM2, murine double minute 2

MODY, maturity-onset diabetes of the young

NEAA, non-essential amino acids solution

one-way ANOVA, one-way analysis of variance

OMIM, Online Mendelian Inheritance in Man

PNDM, permanent neonatal diabetes mellitus

PHHI, persistent hyperinsulinemic hypoglycemia in infancy

PBS, phosphate-buffered saline

PROTAC, proteolysis-targeting chimera

R^2, coefficients of determination (R^2)

TCA cycle, tricarboxylic acid cycle

TCGA, The Cancer Genome Atlas

UDP-glucose, uridine diphosphate glucose

UGGT, UDP-glucose:glycoprotein glucosyltransferase

UGP2, UDP-glucose pyrophosphorylase 2

UPR, unfolded protein response

VHL, von Hippel-Lindau

WB, Western blot

WT, wild type

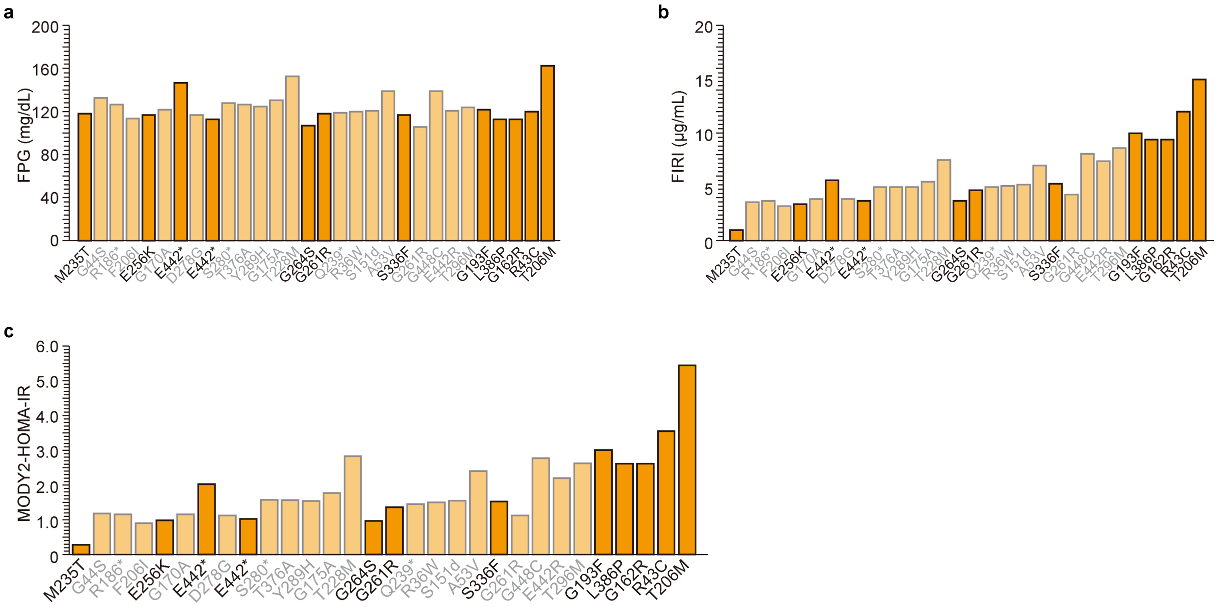

Supplementary Figure S1. Clinical data in MODY2 patients among MODY1-3 (Supplementary data for Fig. 3).

Clinical summary of fasting plasma glucose (FPG mg/dL, **a**), fasting immunoreactive insulin (FIRI, µg/mL, **b**), and homeostatic model assessment for insulin resistance (HOMA-IR, **c**) in MODY2 patients. Eleven glucokinase mutated proteins in strong orange bars (M235T, E256K, E442*, G264S, G261R, S336F, G193F, L388P, G162R, R43C, and T206M) were analyzed *in vitro*.

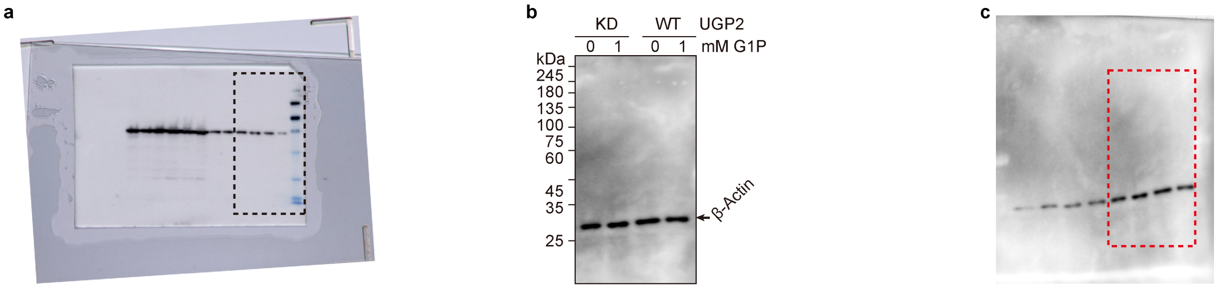

Supplementary Figure S2. Low glucose induced UDP-glucose-dependent glucokinase ubiquitination and degradation (Supplementary data for Figure 1).

The administration of G1P had no discernible impact on the quantity of glucokinase protein.

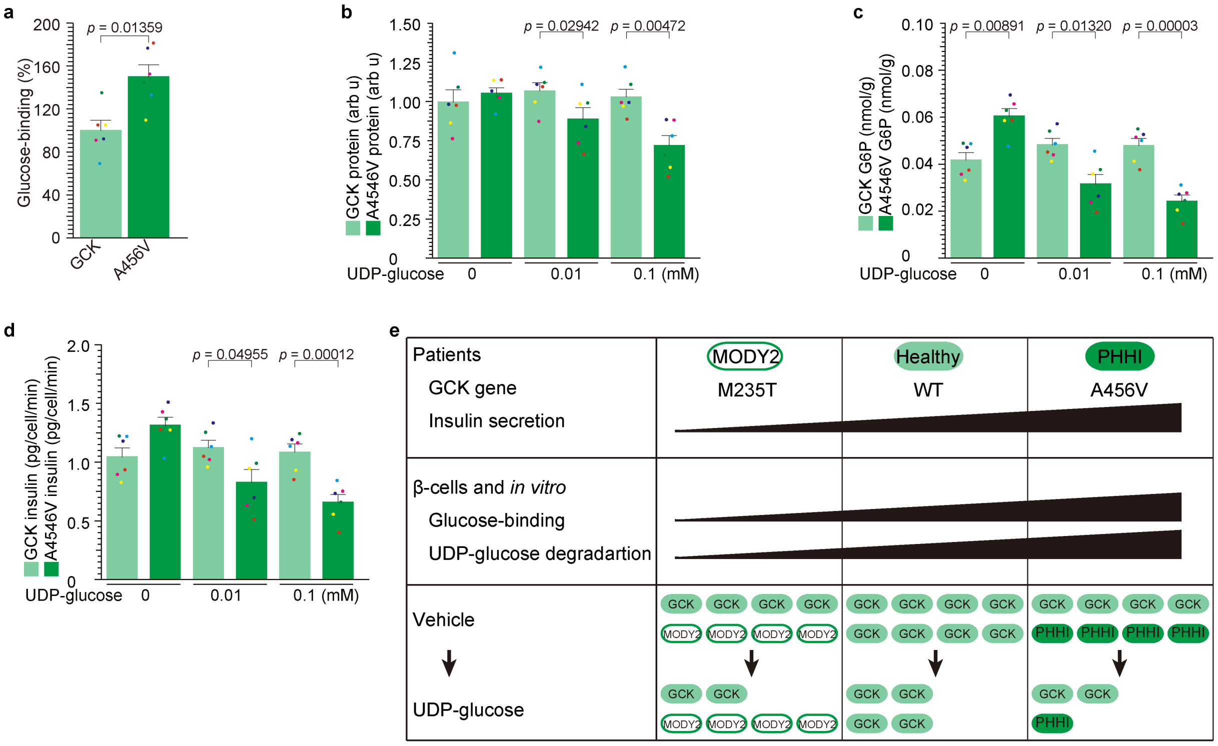

Supplementary Figure S3. Optimizing Substrate Protein Glucokinase for UDP-Glucose-Dependent Ubiquitination and Degradation (Supplementary data for Fig. 3).

(a) The A456V glucokinase variant from PHHI exhibited a strong binding affinity for glucose. The glucose-binding activity of glucokinase WT (GCK) and A456V (PHHI) was assessed using glucose-immobilized beads [1].

(b –d) A456V demonstrated greater sensitivity to UDP-glucose-dependent glucokinase protein degradation (b), G6P production (c), and insulin secretion (d). UDP-glucose-dependent GCK and A456V degradation (**b**), G6P production (**c**), and insulin secretion (**d**) were analyzed.

MODY2変異	MODY2					MODY2		
	FPG	FIRI	HOMA-B	HOMA-R	I.I	初診時HbA1c	BMI	onset age
GCK A53V	139	7	33.1578947	2.40246914	0.38	6.2	14.6	11
GCK T209R	121	14			0.49	6.7	19.7	10
GCK K56T	.	.			.	.	.	.
K p.S280LfsTe	128	5	27.6923077	1.58024691	0.52	5.9	18.8	14
GCK Gly193Trp	122	10	61.0169492	3.01234568	0.68	6.5	16.2	14
c.1254 IVS9 -1	123	5	30	1.51851852	.	6	21.5	6
GCK E242V	.	.			.	.	17.0	6
GCK M37V	123	.			0.17	5.7	.	11
e348AsnとPro	145	2.6	11.4146341	0.9308642	0.1	7	14.8	0
GCK A188T	.	.			.	.	.	.
exon1のdelet	.	.			.	.	18.2	16
GCK:IVS1+1G>T	.	.			.	6.3	20.4	6
GCK A379E	.	.			.	6.2	.	0
delCAGAATG; p	119	4.97	31.95	1.46032099	0.58	5.7	17.8	18
GCK E442Ter	113	3.7	26.64	1.03234568	0.44	5.3	20.1	22
全エクソンdele	122	8.88	54.1830508	2.67496296	.	6.5	16.3	15
GCK Arg43Cys	120	12	75.7894737	3.55555556	0.54	6.1	15.4	10
GCK G261E	109	2.45	19.173913	0.65938272	.	6.2	14.4	7
c.863 IVS7 +1	.	.			.	6.1	18.1	5
GCK Gly264Ser	107	3.7	30.2727273	0.97753086	0.32	5.9	21.1	19
GCK:Met235Thr	118	0.97	6.34909091	0.28261728	0.18	6.4	14.4	6
GCK M381R	.	.			.	7	22.9	18
GCK:Gly261Arg	.	.			.	5.8	13.4	2
Thr376_Ala37	127	5	28.125	1.56790123	0.24	6.7	13.8	8
GCK:Gly261Arg	118	4.7	30.7636364	1.36938272	0.72	6.7	14.6	13
GCK G170A	122	3.87	23.6135593	1.16577778	0.3	5.8	17.5	25
GCK L386P	113	9.4	67.68	2.62271605	0.56	6.2	22.7	8
GCK R358Ter	126	.			.	6.2	15.5	10
GCK Asn254Ser	123	3.3	19.8	1.00222222	.	6.2	18.8	5
Glu442ArgfsTe	121	7.4	45.9310345	2.2108642	0.53	6.6	18.4	23
GCK Gly44Ser	133	3.6	18.5142857	1.18222222	0.29	6.4	14.2	8
GCK Thr206Met	124	8.6	50.7540984	2.63308642	0.67	6.3	17.1	11
GCK R186Ter	127	3.7	20.8125	1.16024691	0.16	7.4	14.6	10
GCK Y289H	125	5	29.0322581	1.54320988	0.2	6.8	18.4	8
GCK Ser151del	121	5.2	32.2758621	1.55358025	0.2	6.6	18.0	3
K D187EfsTer	138	7.3	35.04	2.48740741	.	7.4	16.8	12
GCK D278G	117	3.89	25.9333333	1.12377778	0.55	6.6	19.2	14
GCK A188T	.	.			.	6.7	17.8	12
GCK G175A	131	5.5	29.1176471	1.77901235	0.14	6.7	22.4	8
GCK G162R	113	9.4	67.68	2.62271605	0.56	6.4	22.7	8
GCK F260I	114	3.2	22.5882353	0.90074074	0.41	6.8	21.9	5
GCK Pro59Ser	134	5	25.3521127	1.65432099	.	7.6	15.2	5
CK Ser336Tr	117	5.3	35.3333333	1.53111111	0.47	7.6	20.1	19
GCK A387V	.	.			.	6.5	18.7	3
GCK ex1a欠損	113	0.5	3.6	0.13950617	0.3	6.4	16.0	1
GCK G261R	106	4.3	36	1.1254321	0.29	6.6	14.1	11
GCK Q239R	132	2.5	13.0434783	0.81481481	.	9.4	22.1	13
GCK E442Ter	126	6.5	37.1428571	2.02222222	.	6.8	18.2	29
T228M	153	7.5	30	2.83333333	0.6	6.7	15.4	14
GCK ex1a欠損	116	10.3	69.9622642	2.95012346	.	6.5	17.3	14

A370P	115	.			.	6.5	19.4	21
GCK M381R	132	5.1	26.6086957	1.66222222	.	6.6	18.3	4
GCK R369P	114	3.3	23.2941176	0.92888889	.	6.4	19.1	35
GCK T206M	163	38.4	138.24	15.4548148	.	6.8	19.2	13
Tyr289MetfsT	183	.			.	6.8	21.3	13
GCK V253F	131	.			.	6.6	22.4	37
GCK L58P	133	9.3	47.8285714	3.05407407	.	6.3	15.0	6
GCK S375P	122	3.3	20.1355932	0.99407407	.	6.8	16.3	9
GCK R36W	120	5.1	32.2105263	1.51111111	0.32	6.3	21.4	17
GCK A188T	91	.			.	6.4	20.5	25
GCK M235T	141	.			.	6.7	15.4	5
GCK G448C	139	8.1	38.3684211	2.78	0.36	5.8	20.5	26
GCK E256K	117	3.4	22.6666667	0.98222222	.	6.9	19.4	13
GCK E442Ter	147	5.6	24	2.03259259	0.27	6.7	15.0	10
GCK Y296Ter	115	.			.	6.7	15.7	6
·CK IVS7+1 G>'	122	4.3	26.2372881	1.29530864	.	6.7	13.6	6
